# Invasive Fungal Infections after Natural Disasters

**DOI:** 10.3201/eid2003.131230

**Published:** 2014-03

**Authors:** Kaitlin Benedict, Benjamin J. Park

**Affiliations:** Centers for Disease Control and Prevention, Atlanta, Georgia, USA

**Keywords:** fungus, fungal infection, disasters, tsunamis, tornadoes, injuries, pneumonia, meningitis, mucormycosis, coccidioidomycosis, fungi

## Abstract

The link between natural disasters and subsequent fungal infections in disaster-affected persons has been increasingly recognized. Fungal respiratory conditions associated with disasters include coccidioidomycosis, and fungi are among several organisms that can cause near-drowning pneumonia. Wound contamination with organic matter can lead to post-disaster skin and soft tissue fungal infections, notably mucormycosis. The role of climate change in the environmental growth, distribution, and dispersal mechanisms of pathogenic fungi is not fully understood; however, ongoing climate change could lead to increased disaster-associated fungal infections. Fungal infections are an often-overlooked clinical and public health issue, and increased awareness by health care providers, public health professionals, and community members regarding disaster-associated fungal infections is needed.

The potential for adverse health events after natural disasters is well recognized and comprises various challenges for public health (*1*). The World Health Organization defines a disaster as a disruption of society resulting in widespread human, material, or environmental loss that exceeds the affected society’s ability to cope by using local resources (*2*). Natural disasters can be broadly classified into 3 groups: geophysical (e.g., earthquakes, volcanic eruptions, and tsunamis), hydrometeorological (e.g., floods, hurricanes, and tornadoes), and geomorphological (e.g., landslides and avalanches). Specific social, economic, and cultural settings create a unique set of circumstances for every disaster, and the immediate causes of illness and death (such as blunt trauma, lacerations, crush injuries, suffocation, and drowning) vary according to the type of event (*1*).

Infectious disease outbreaks after natural disasters are uncommon. However, features of the post-impact and recovery phases of disasters, such as population displacement, low vaccine coverage for vaccine-preventable diseases, inadequate sanitation and hygiene infrastructure, and limited access to health care services, can interact to increase the risk for transmission of infectious diseases that were previously established in the affected area (*3*). Disaster-associated fungal infections are similarly uncommon, but they are becoming increasingly recognized and are typically attributable to the impact phase of a disaster because such infections primarily result from inhalation or cutaneous inoculation of fungal spores directly from the environment (*3*,*4*). During a disaster, pathogenic fungi can be displaced from their natural habitats, which could increase their environmental concentration or introduce them to areas where they would not normally be found, resulting in contact with injured persons and potentially causing fungal infections. To increase awareness of these events among health care providers and public health officials, we summarize the known occurrences of fungal infections associated with natural disasters (Table).

**Table:** Disaster-associated fungal infections*

Disaster	Reference	Location	No. cases	Fungal organism	Type of infection	Outcome
Tornado, 2011	Neblett Fanfair et al. (*4*)	USA	13	*Apophysomyces trapeziformis*	Soft tissue	38% all-cause mortality
Great East Japan Earthquake and Tsunami, 2011	Kawakami et al. (*5*)	Japan	1	*Aspergillus fumigatus*	Pulmonary, multi-organ dissemination	Death
	Nakamura et al. (*6*)	Japan	1	*Scedosporium apiospermum*	Lung and brain abscesses	Death
	Igusa et al. (*7*)	Japan	1	Pathogen not identified†	Sinusitis and meningitis	Death
Hurricane Ike, 2008	Riddel et al. (*8*)	USA	3	Unspecified agent of chromoblastomycosis	Soft tissue	Recovery
Hurricane Katrina, 2005	Rao et al. (*9*)	USA	1	*Cladosporium* sp.	Pulmonary	Resolved without treatment
Indian Ocean Tsunami, 2004	Petrini et al. (*10*)	Thailand	2	*Cladophialophora bantiana*	Soft tissue	Recovery
	Garzoni et al. (*11*	Thailand	2	*Scedosporium apiospermum*	Spondylodiscitis, 1; brain abscess,1	Recovery
	Gunaratne et al. (*12*)	Colombo, Sri Lanka	6	*A. fumigatus*	Meningitis	50% all-cause mortality
	Andresen et al. (*13*)	Sri Lanka	1	*Apophysomyces elegans*	Soft tissue	Not specified
	Snell and Tavakoli (*14*)	Thailand	1	*A. elegans*	Soft tissue	Recovery
	Maegele et al. (*15*)	Southeast Asia	1	*Fusarium* sp.	Soft tissue, sepsis	Death
			1	*Mucor* sp.	Soft tissue	Not specified
Earthquake, 1994	Schneider et al. (*16*)	USA	203	*Coccidioides immitis*	Pulmonary; 6 (3.7%) disseminated	1.5% all-cause mortality
Volcano, 1985	Patiño et al. (*17*)	Colombia	8	*Rhizopus arrhizus*	Soft tissue	80% all-cause mortality
Dust storm originating near Bakersfield, California, 1977	Flynn et al. (*18*)	USA	115	*C. immitis*	Pulmonary; 16 (14%) disseminated	7% all-cause mortality
	Williams et al. (*19*)	USA	18	*C. immitis*	Pulmonary; 4 (22%) disseminated	5.5% all-cause mortality

*As documented in reports with sufficient detail about the number of persons affected, pathogen, and body site. Reports describing cases of post-disaster fungal colonization without infection are not included in the table. Number of cases and percentages are provided when data were available. †Fungal infection diagnosed on the basis of cerebrospinal fluid profile (decreased glucose, high mononuclear cell count, + β-D glucan test result).

## Search Strategy and Selection Criteria

The online literature databases PubMed and Google Scholar were searched for English-language articles about fungal infections related to natural disasters that were published as of April 2013. Search terms included combinations of “disaster,” “natural disaster,” “tornado,” “hurricane,” “earthquake,” “tsunami,” and “flood” with “fungal infection,” “fungal disease,” “fungus,” or “mold.” References cited in relevant articles were also reviewed, and the authors’ personal records were searched for conference abstracts.

## Pathogenic Fungi and the Environment

Of the 1.5 million species of fungi on Earth, ≈300 are known human pathogens (*20*). Pathogenic fungi exist in a broad range of natural habitats but are believed to be more common in subtropical and tropical areas of the world, probably because of environmental restrictions on their growth or propagation (*20*). Known geographic habitats of some pathogenic fungi (for example, *Blastomyces*, *Coccidioides*, and *Histoplasma*) are well defined, but others (such as *Aspergillus* and other molds) are thought to be ubiquitous. The abundance and distribution (and therefore, potential to cause disease) of environmental fungi probably depend on climatic or environmental factors such as ambient temperature and moisture (*20*). Examples include coccidioidomycosis incidence in Arizona, which has been shown to correlate with hot, dry conditions; blastomycosis clusters observed in association with rainfall after periods of decreased precipitation; and incidence of *Penicillum marneffei* penicilliosis, an opportunistic infection which is endemic to Southeast Asia and increases in incidence during rainy months (*21*,*22*). Aspergillosis and other invasive mold infections have also been postulated to correlate with seasons or weather patterns in some geographic areas (*23*).

Although seasonal changes and weather effects probably play a role in the growth and distribution of many pathogenic fungi, environmental disruption is a key factor in the dispersal of these organisms and their resulting potential for causing infection. Both small-scale earth-disrupting activities, such as excavation or construction, and events affecting larger areas, such as earthquakes (*16*), tsunamis (*5*–*7*,*10*–*15*,*24*,*25*), and tornadoes (*4*), have been linked to the occurrence of fungal infections. Natural disasters can cause large-scale disruption of fungal habitats, which can lead to clusters of respiratory, cutaneous, or other forms of fungal disease.

Studies of clinical specimens collected from persons injured during disasters highlight the diversity of potential fungal pathogens in the natural environment. For example, after an 8.0-magnitude earthquake in Wenchuan, China, during 2008, 19 strains of fungi were identified in wound, sputum, and blood cultures from 123 injured persons (*26*), and fungi accounted for 7.6% of clinical isolates obtained from 42 patients with crush syndrome (*27*). Similarly, after a 1970 tornado in Texas, United States, fungi were identified in 8 (6.5%) of 124 wound isolates from 24 hospitalized patients (*Fusarium*, unspecified yeast, *Rhodotorula*, *Aspergillus*, *Hormodendrum* [now *Cladosporium*], and *Cephalosporium*), and in 4 (10.5%) of 38 wound isolates from 23 ambulatory patients (*28*). In each of these reports, multiple organisms were isolated from many of the patients, and the fungi recovered may not necessarily have been agents of infection.

## Respiratory Fungal Infections after Disasters

### Airborne

Inhalation is a common route for fungal infections. Fungi are known to cause respiratory infections ranging from asymptomatic to life-threatening, depending on the pathogen and host characteristics. *Coccidioides* spp. are dimorphic fungi that grow in semiarid soil and are endemic to the southwestern United States, northern Mexico, and parts of South America. Two instances of disaster-associated coccidioidomycosis have been described.

An outbreak of coccidioidomycosis after the January 1994 earthquake in Northridge, California, United States, was 1 of few known examples of any infectious disease outbreak directly related to a geophysical disaster (*3*). *Coccidioides* spores were presumably aerosolized as a consequence of the earthquake, its aftershocks, and associated landslides and were dispersed by the resulting widespread dust clouds (*16*). In Ventura County, California, 203 outbreak-associated coccidioidomycosis cases were identified, and investigators found that dust exposure was substantially associated with acute illness (*16*). Fungal infection may not have been considered in the initial diagnoses in this outbreak; 93% of case-patients received >1 antibacterial drug before coccidioidomycosis was diagnosed (*16*).

Another coccidioidomycosis outbreak occurred after a severe dust storm in the southern San Joaquin Valley of California in December 1977 (*18*). The storm originated near Bakersfield, an area to which coccidioidomycosis is highly endemic, and covered nearly 90,000 km^2^, an area larger than the state of Maine (*18*). In Sacramento County, an area to which the disease was not previously considered to be endemic, 115 cases of coccidioidomycosis were attributed to the dust storm, including 16 cases of disseminated disease (*18*). Eighteen additional cases were identified at a US Navy air station in Kings County (*19*), and other California counties affected by the storm saw more coccidioidomycosis cases than usual; for example, Kern County recorded 134 cases during January and February 1978, compared with 17 cases during those months in the previous year (*29*).

### Near-drowning

Drowning and near-drowning are common during and after disaster-related flooding (*1*). Aspiration of contaminated or debris-laden water can lead to sinus and pulmonary infections; aspiration pneumonia is often referred to as “tsunami lung” in post-tsunami settings (*16*,*24*). Tsunami lung can be caused by bacteria, fungi, or both. *Pseudallescheria boydii* (asexual form*, Scedosporium apiospermum*) is hypothesized to be the most common fungal pathogen associated with near-drowning, although this finding has not been studied specifically in the context of disasters (*30*). Information about post-disaster *Scedosporium* lung infection is limited to a small number of case reports; these reports also document the organism’s propensity to progress to central nervous system infection, even in immunocompetent hosts (*6*,*11*). Other fungal pathogens, such as *Aspergillus*, have also been implicated as agents of tsunami lung; after the 2011 Great East Japan Earthquake and subsequent tsunami, a previously healthy near-drowning victim who later died was found to have pneumonia caused by *Aspergillus fumigatus* and evidence of multiorgan disseminated aspergillosis upon autopsy (*5*). The report describes delays in specimen transportation and receipt of culture results caused by the aftermath of the earthquake, which led to a delay in diagnosis and treatment (*5*).

Evidence of tsunami lung also was also apparent after the December 2004 earthquake and tsunami in the Indian Ocean, which killed >200,000 persons (*3*). In Sri Lanka, acute respiratory issues attributed to post-aspiration pneumonitis and polymicrobial pneumonia that were not related to communicable illnesses were the most frequent medical problems after this disaster (*31*). In Banda Aceh, Indonesia, several patients with necrotizing pneumonia, who did not respond to broad-spectrum antibacterial drugs, probably had polymicrobial infections that may have included fungal organisms (*24*). However, limited diagnostic capacity for fungi may have affected the ability to identify the potential role of fungi in these infections. A report from Germany demonstrated that among a cohort of 17 tourists injured during the tsunami, all had clinical and radiologic evidence of aspiration pneumonitis and pneumonia; *Candida albicans* and *A. fumigatus* were isolated from the respiratory tract of several patients, although it is unclear whether the isolation of these organisms represented true infection or colonization (*15*).

## Soft Tissue Fungal Infections after Disasters

The risk for wound infections after a natural disaster is high when wounds are contaminated with water, soil, or debris (*32*). In addition, damage to the local health care infrastructure can compromise the ability to properly irrigate contaminated wounds with sterile solution or promptly treat injured persons with topical or systemic antimicrobial drugs (*32*). These factors can result in severe, often polymicrobial, infections of otherwise relatively minor injuries (*32*). Although most documented disaster-associated soft tissue infections are bacterial (typically gram-negative pathogens such as *Aeromonas*, *Escherichia coli*, and *Klebsiella*) (*33*), fungal wound infections can also occur, and they could be under-recognized because they can be clinically similar to bacterial infections, particularly during the early stages of infection.

Mucormycosis, caused by fungi that belong to the order Mucorales, is perhaps the most recognized example of post-disaster fungal soft tissue infection. Necrotizing fasciitis can result, and case-fatality rates of ≈30% are frequently described, although early diagnosis and treatment has been shown to lead to better outcomes (*34*). The first documented instance of disaster-associated mucormycosis occurred after the 1985 volcanic eruption in Armero, Colombia, which caused an estimated 23,000 deaths and ≈4,500 injuries (*17*). According to a report of 38 patients with necrotizing lesions who were hospitalized after the volcano, 8 patients had infections caused by the mucormycete *Rhizopus arrhizus* (*oryzae*) (*17*).

Similarly, a cluster of mucormycosis cases caused by *Apophysomyces trapeziformis* occurred among 13 persons who were severely injured in the May 22, 2011, tornado in Joplin, Missouri, United States (*4*). Penetrating trauma and an increased number of wounds were shown to be independent risk factors for mucormycosis. Whole-genome sequencing of *A. trapeziformis* isolates from case-patients’ wounds (Figure) showed 4 nonidentical but closely related strains of *A. trapeziformis*. This finding, considered with case-patients’ receipt of medical care at different hospitals, suggested that the infections were acquired from the natural environment as a result of exposure to organic matter and water, which are likely reservoirs for mucormycetes (*4*).

**Figure:**
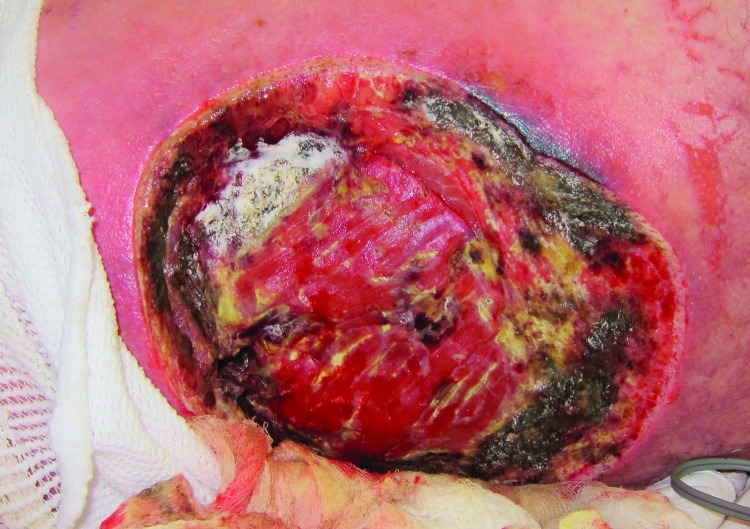
Necrotizing cutaneous mucormycosis, Joplin, Missouri, USA, 2011 (*4*). A left flank wound in a mucormycosis case-patient, with macroscopical fungal growth (tissue with white, fluffy appearance) and necrotic borders before repeated surgical debridement. Copyright 2012 Massachusetts Medical Society. Reprinted with permission.

In addition to the 2 clusters described, several isolated cases of post-disaster soft tissue mucormycosis have been reported, notably among persons injured during the 2004 Indian Ocean tsunami (*13*–*15*). These reports illustrate some of the clinical challenges associated with soft tissue mucormycosis, caused by organisms that may initially appear indistinguishable from other types of wound infections but require aggressive treatment with intravenous antifungal medication and surgical debridement (*13*,*14*).

Other agents of fungal soft tissue infections in survivors injured during the 2004 Indian Ocean tsunami include *Fusarium* (which later caused systemic infection) in 1 tourist (*15*) and *Cladophialophora bantiana* in 2 other tourists (*10*). Subcutaneous *C. bantiana* infection has also been associated with a tornado-associated injury in which the patient was inoculated by a contaminated wood splinter but did not have symptoms until ≈16 years later (*35*). Soft tissue fungal infections have also been documented among persons who were not directly injured during a disaster but who sustained minor trauma while performing post-disaster tasks: in Texas after Hurricane Ike in 2008, chromoblastomycosis was diagnosed in 3 patients, all of whom had histories of cancer and all of whom described clearing brush and fallen trees near their homes after the storm (*8*).

## Health Care–associated Fungal Infections after Disasters

Although respiratory and cutaneous infections are the most commonly described forms of fungal infection after natural disasters, other, more invasive fungal infections have also been observed. An outbreak of *Aspergillus* meningitis after the 2004 Indian Ocean tsunami was associated with the use of spinal anesthesia for cesarean section infant delivery for 6 previously healthy women in Sri Lanka (*12*). The first 5 case-patients were initially treated for bacterial meningitis, but the discovery of *Aspergillus* during the post-mortem examination of the index case-patient led to the use of amphotericin B and voriconazole in the surviving case-patients (*12*). Investigation of various medical supplies revealed that syringes from a central storage facility were contaminated with *A. fumigatus*, probably as a result of suboptimal storage conditions in a humid warehouse (*12*).

## Indoor Mold Exposures after Disasters

Disaster-induced water damage to structures can create moist environments that can promote indoor fungal growth, but the extent to which damp indoor spaces and mold growth affect human health remains somewhat ambiguous (*36*). A 2004 report by the Institute of Medicine found sufficient evidence of association between indoor mold exposure and upper respiratory tract symptoms, cough, and wheezing, and evidence of an association between indoor mold exposure and some noninfectious health conditions that included asthma symptoms in persons with asthma and hypersensitivity pneumonitis in some groups of people (*36*). Although the report found no association between indoor mold exposure and invasive infection in healthy persons, there was evidence to support a link between exposure to *Aspergillus* and aspergillosis in severely immunocompromised persons (*36*).

Few data clearly demonstrate that indoor mold exposures increase the risk for invasive infection in post-disaster settings. Flooding lasted for weeks after Hurricanes Katrina and Rita made landfall on the US Gulf Coast in August and September 2005, respectively, leading to visible mold growth in 46% of 112 inspected homes (*37*). Despite these high levels of indoor mold growth documented in some areas, 1 study showed no elevated risk for fungal infections among immunocompromised patients exposed to water-damaged buildings after Hurricane Katrina; 1 patient, 1.2% of the profoundly immunocompromised study population, had a mold infection (caused by a *Cladosporium* sp.), which resolved without antifungal treatment (*9*). Colonization in the absence of related clinical symptoms was observed in persons who returned to their water-damaged homes after Hurricanes Rita and Katrina: the mucormycete *Syncephalastrum* was detected in various clinical specimens from 8 persons whose self-reported exposures to mold ranged from none to heavy, but none had evidence of invasive infection (*38*).

After the 2011 Great East Japan Earthquake and subsequent tsunami, a medical relief team observed unexplained chronic cough among a group of previously healthy persons living in a temporary refuge (*25*). Fungal cultures of sputum samples from 6 persons yielded *Aspergillus fumigatus*, *A. flavus*, and basidiomycetous fungi; culture plates exposed inside the refuge showed a similar fungal profile, suggesting that the indoor environment may have played a role in the patients’ infections (*25*). Although the authors of that report state that the patients’ coughs resolved after treatment with the antifungal itraconazole, the extent to which an infectious process was responsible for the illnesses is unclear (*25*).

## Disasters, Fungi, and Global Climate Change

Climate change could be affecting the ecology of pathogenic fungi in ways that are not yet fully understood; even minor or gradual changes in temperature, moisture, and wind patterns might affect fungal growth, distribution, and dispersal (*20*). For example, warmer average global temperatures may allow the geographic range of fungi typically restricted to tropical and subtropical environments, such as *Cryptococcus gattii*, to expand into areas that are currently more temperate (*20*). Global warming has also been hypothesized to select for fungi with tolerance to warmer temperatures (*20*). The relative scarcity of fungal diseases among mammals has been hypothesized to be associated with the inability of many fungal species to survive at temperatures >37°C; however, warmer ambient temperatures may enable nonpathogenic fungi to acquire the ability to infect warm-blooded hosts (*20*).

Huppert and Sparks suggest that global climate change is contributing to greater frequency and severity of extreme weather events and that current patterns of population growth, urbanization, and human activity create conditions that render many communities increasingly vulnerable to these hazards (*39*). Coupled with an increased risk for natural disasters, a larger or more geographically widespread ecologic burden of pathogenic fungi could lead to greater numbers of disaster-associated fungal infections through any of several mechanisms: inhalation of spores dispersed as a result of geophysical disruption, traumatic implantation of fungi into wounds contaminated with organic matter, or infection associated with suboptimal medical care where the local health care system has been damaged or destroyed. Altogether, a combination of factors including genetic and biological aspects of host–pathogen interactions; changing features of the physical environment; and social, political, or economic influences could lead to the emergence of new fungal pathogens or increased numbers of infections by known pathogens (*40*).

## Conclusions

Disasters are complex events that can result in a wide range of health effects, although infectious disease outbreaks as an immediate consequence of disasters are uncommon. Health care providers should be aware of the possibility for cases or clusters of community-acquired or health care–associated fungal infections among disaster survivors because these infections often appear clinically similar to bacterial infections and can be associated with serious illness and death. These infections can occur in persons who do not have the typical immunocompromising risk factors for fungal infection but who have experienced near-drowning, trauma, or other unusual exposure to the environment, such as a dust storm. A fungal infection should be considered early if a patient has a persistent or progressive infection that is not responding to initial antibacterial treatment, particularly because rapid diagnosis and administration of appropriate antifungal therapy can improve patient outcomes. Prompt restoration of disaster-affected aspects of the local health care infrastructure may help facilitate earlier diagnosis and treatment and possibly reduce the risk for infection associated with the use of contaminated medical equipment or substandard care. Strategies to reduce disaster-associated fungal infections should be considered within the broader context of comprehensive and sustainable risk reduction methods to prevent disaster-related injury and illness.
